# Asymmetrical cortical vessel sign predicts prognosis after acute ischemic stroke

**DOI:** 10.1002/brb3.1657

**Published:** 2020-05-20

**Authors:** Yong‐Lin Liu, Wei‐Min Xiao, Jie‐Kai Lu, Ya‐Zhi Wang, Zhi‐Hao Lu, Huo‐Hua Zhong, Jian‐Feng Qu, Xue‐Wen Fang, Man‐Qiu Liang, Yang‐Kun Chen

**Affiliations:** ^1^ Department of Neurology Dongguan People's Hospital (Affiliated Dongguan Hospital, South Medical University) Dongguan China; ^2^ Department of Radiology Dongguan People's Hospital (Affiliated Dongguan Hospital, South Medical University) Dongguan China

**Keywords:** acute ischemic stroke, asymmetrical cortical vessel sign, intravenous thrombolysis, outcome, susceptibility‐weighted imaging

## Abstract

**Introduction:**

To assess whether the asymmetrical cortical vessel sign (ACVS) on susceptibility‐weighted imaging (SWI) could predict 90‐day poor outcomes in anterior circulation acute ischemic stroke (AIS) patients treated with recombinant tissue plasminogen activator (r‐tPA).

**Methods:**

Clinical data of consecutive patients with anterior circulation AIS treated with r‐tPA were retrospectively analyzed. Clinical variables included age, sex, vascular risk factors, NIHSS score, onset to treatment time, and initial hematologic and neuroimaging findings. Follow‐up was performed 90 days after onset. Poor outcome was defined as a modified Rankin scale (mRS) ≥3 at 90 days.

**Results:**

A total of 145 patients were included, 35 (24.1%) patients presented with ACVS (≥Grade 1) on SWI. Fifty‐three (36.6%) patients had a poor outcome at 90 days. ACVS (≥Grade 1) occurred in 21 (39.6%) patients with poor outcome compared with 14 (15.2%) patients with favorable outcome (*p* = .001). Univariate analysis indicated that age, NIHSS score on admission, previous stroke, hemorrhagic transformation, severe intracranial large artery stenosis or occlusion (SILASO), and ACVS were associated with 90‐day poor outcome (*p* < .05). Since SILASO and ACVS were highly correlated and ACVS had different grades, we used three logistic regression models. Results from the three models showed that ACVS was associated with 90‐day poor outcome.

**Conclusions:**

In r‐tPA‐treated patients with anterior circulation AIS, ACVS might be a helpful neuroimaging predictor for poor outcome at 90 days.

## Graphical abstract

To the best of our knowledge, this is the first study to evaluate the effects of asymmetrical cortical vessel sign (ACVS) on predicting clinical outcomes in patients with acute ischemic stroke (AIS) after intravenous thrombolysis (IVT) therapy. ACVS might be a helpful neuroimaging predictor for 90‐day poor outcome in AIS patients treated with IVT. This finding may help physicians to identify patients at relatively high risk of poor prognosis.

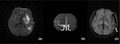

## 1 INTRODUCTION

Intravenous thrombolysis (IVT) with recombinant tissue plasminogen activator (r‐tPA) is an effective treatment for acute ischemic stroke (AIS) patients when administered within 4.5 hr of stroke onset (Hacke et al., 2008; The National Institute of Neurological Disorders & Stroke RT‐PA Stroke Study Group, 1995). However, only about 50% (45.0%–52.4%) of patients treated with r‐tPA had a favorable outcome in previous studies (Hacke et al., 2008; Lees et al., 2010; Wahlgren et al., 2008). Several clinical factors were confirmed as predictors of long‐term outcome, including higher National Institutes of Health Stroke Scale (NIHSS) score on admission (Adams et al., 1999), older age (Nakayama, Jørgensen, Raaschou, & Olsen, 1994), and longer onset to treatment time (OTT) (Marler et al., 2000). Magnetic resonance imaging (MRI) predictors included lesion topography (Liu et al., 2015) and presence of the M1 susceptibility vessel sign (Kimura et al., 2009). There is increasing evidence that indicates perfusion status in ischemic brain regions is related to outcomes in AIS patients after IVT (Bivard et al., 2015; Kawano et al., 2017; Kim, Kim, Oh, & Kim, 2004). Susceptibility‐weighted imaging (SWI), an MRI sequence that is highly sensitive to paramagnetic materials such as deoxyhemoglobin and hemosiderin (Huang et al., 2012; Miyasaka et al., 2012), can provide indirect information on regional perfusion status (Kao, Tsai, & Hasso, 2012). In recent years, the novel SWI‐MRI marker asymmetrical cortical vessel sign (ACVS) has been associated with poor outcomes in anterior circulation AIS patients who had not undergone IVT therapy (Sun et al., 2014; Wang et al., 2018). However, to our knowledge, there has been no study of ACVS in patients following IVT. Therefore, we evaluated the effects of ACVS on clinical outcomes in anterior circulation AIS after IVT therapy.

## 2 METHODS

### 2.1 Patients

Acute ischemic stroke patients treated with r‐tPA IVT admitted to Dongguan People's Hospital between 1 January 2016 and 31 December 2018 were consecutively recruited. The inclusion criteria were as follows: (a) age >18 years, (b) MRI‐confirmed AIS of anterior circulation, (c) onset of stroke symptoms within 4.5 hr and treatment with r‐tPA, (d) prestroke modified Rankin Scale (mRS) score ≤1, and (e) MRI‐SWI examination performed. The exclusion criteria were as follows: (a) no acute lesion found on diffusion‐weighted imaging (DWI), (b) AIS of posterior circulation confirmed by MRI, and (c) additional endovascular therapy after IVT. This study was approved by the hospital ethics committee (approval number: KYKT2018‐002). The consent of all subjects was obtained in accordance with the Declaration of Helsinki.

### 2.2 Clinical data collection

NIHSS and OTT values were collected, as well as demographic data including age, sex, history of hypertension, diabetes mellitus, smoking, atrial fibrillation, and previous stroke. Stroke subtype was classified according to the Trial of Org 10172 in Acute Stroke Treatment (TOAST) criteria (Adams et al., 1993) by each patient's attending neurologist.

### 2.3 Clinical outcome

We followed up these patients and assessed their mRS at 90 days during a face‐to‐face interview or via telephone. Favorable and poor outcomes were defined as mRS ≤2 and ≥3, respectively. Recurrence of stroke and death within the follow‐up period were also recorded.

### 2.4 MRI analysis

Brain MRI including T1‐weighted imaging (T1WI), T2‐weighted imaging (T2WI), fluid‐attenuated inversion recovery (FLAIR), DWI, SWI, and three‐dimensional time‐of‐flight magnetic resonance angiography (3D‐TOF‐MRA) was performed for each participant using a 3.0 T system (Skyra; Siemens Medical) within 72 hr after admission.

Axial SE T1 (time of repetition [TR]/time of echo [TE]/excitation = 1,500/11/1, field of view [FOV] = 220 mm, slice thickness/gap = 4 mm/1.2 mm, matrix = 320 × 320, time of acquisition = 1 min 26 s) and turbo spin echo (TSE) T2 (TR/TE/excitation = 4,720/96/2, turbo factor 15, FOV = 220 mm, slice thickness/gap = 4 mm/1.2 mm, matrix of 512 × 512, time of acquisition = 1 min 50 s) images were also acquired. Coronal position FLAIR (TR/TE/excitation = 9,000/84/1, FOV = 230 mm, slice thickness/gap = 5 mm/1.5 mm, matrix 320 × 320, time of acquisition = 1 min 50 s) and DWI spin echo planar imaging (EPI; TR/TE/excitation = 4,640/67/1, matrix = 192×192, FOV = 230 mm, slice thickness/gap = 4 mm/1.2 mm, EPI factor = 91, acquisition time = 1 min 44 s) with three orthogonally applied gradients were used, with b values of 0 and 1,000, as well as SWI (TR/TE/excitation = 27/20/1, FOV = 220 mm, slice thickness/gap = 3 mm/0.6 mm, matrix 256 × 256, time of acquisition = 2 min 28 s) and 3D‐TOF‐MRA (TR/TE/excitation = 21/3.42/1, FOV = 200 mm, slice thickness/gap = 0.7 mm/−0.14 mm, matrix 384 × 384, time of acquisition = 3 min 36 s).

Two experienced MRI‐specialized neuroradiologists, who were blinded to the patients’ clinical information, independently evaluated the imaging findings for the presence of ACVS over the ischemic territory.

1. Intracranial large artery stenosis (ILAS) or occlusion was evaluated by3D‐TOF‐MRA. Symptomatic severe ILAS was defined as that affecting the internal carotid artery (ICA) or M1 segment of middle cerebral artery (MCA) ipsilateral to the infarction with >70% diameter loss. Symptomatic intracranial large artery occlusion was defined as signal loss of distal blood flow ipsilateral to the infarction. Intracranial stenosis or occlusion was assessed using the Warfarin Aspirin Symptomatic Intracranial Disease criteria (WASID Study Group, 1998).
2. ACVS was defined as one hemisphere having more and/or larger vessels with greater signal loss than the other on the minimum‐intensity SWI projection (Baik et al., 2012). According to a published study (Verma et al., 2014), ACVS was categorized into four grades as follows: 0, normal appearance of cortical veins (CV); 1, slightly increased caliber of CV; 2, moderately increased caliber of CV; and 3, distinctly increased caliber of CV.
3. Hemorrhagic transformation was confirmed based on T1WI and T2WI in addition to SWI.

### 2.5 Statistical analysis

Statistical analyses were conducted using SPSS for Windows (v.20.0; IBM Corp.). Continuous variables with a normal distribution are reported as mean ± *SD* and nonnormally distributed variables as median and interquartile range. Variables were compared using *t* test, Pearson's chi‐square test, or Fisher's exact test, as appropriate. Variables with *p* < .05 in the univariate analysis were included in further binary multivariate logistic regressions. Statistical significance was defined as *p* < .05 (two‐sided).

## 3 RESULTS

### 3.1 Clinical characteristics of patients

**FIGURE 1:**
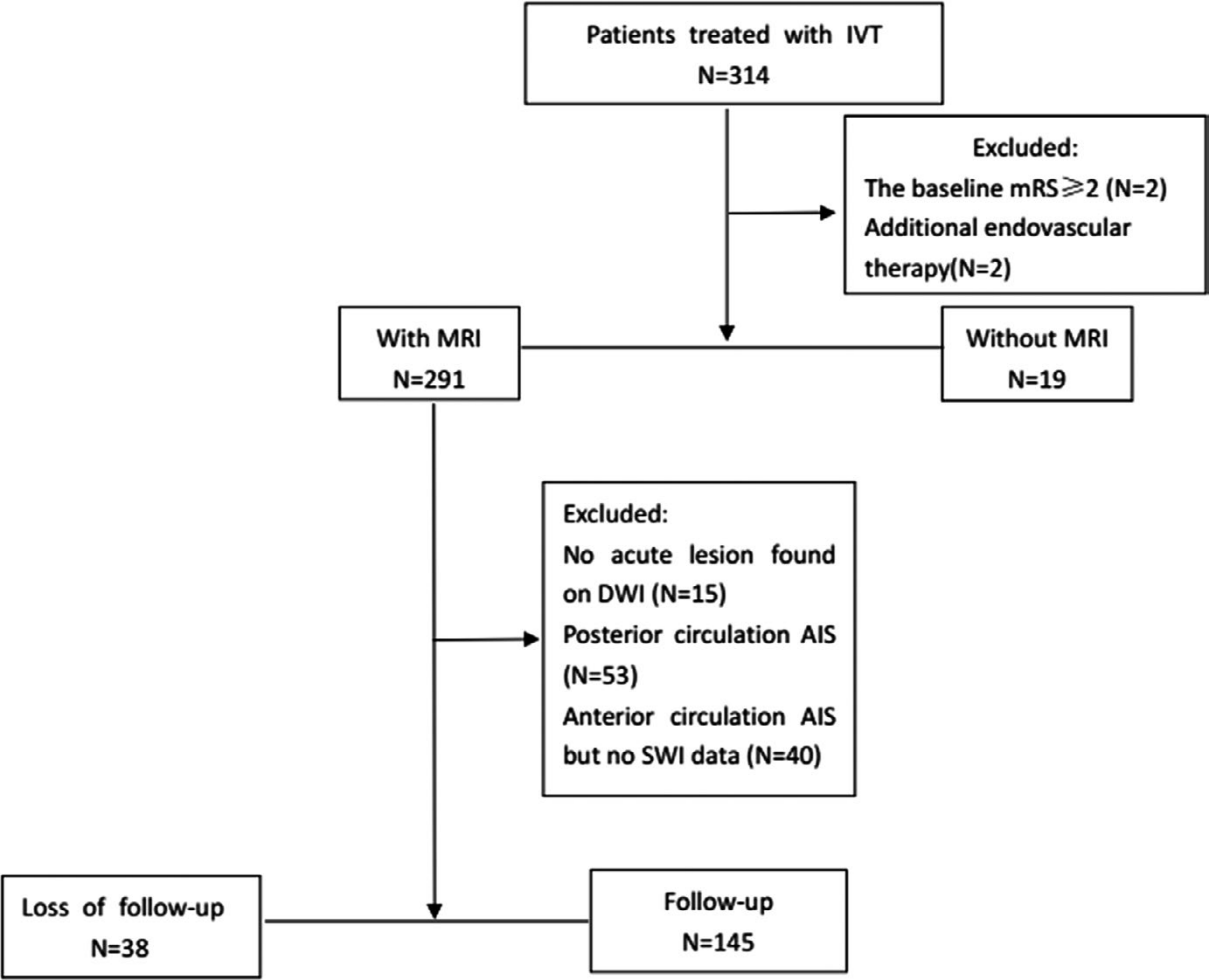
Flowchart of the selection process. AIS, acute ischemic stroke; DWI, diffusion‐weighted imaging; IVT, intravenous thrombolysis; MRI, magnetic resonance imaging; mRS, modified Rankin scale; and SWI, susceptibility‐weighted imaging

During the study period, 314 consecutive patients were treated with r‐tPA IVT within 4.5 hr of stroke onset. In the present study, 169 patients were excluded for the following reasons: additional endovascular therapy after IVT (*n* = 2), baseline mRS ≥ 2 (*n* = 2), permanent or temporary contraindication for MRI (*n* = 19), no acute lesion found on DWI (*n* = 15), posterior circulation AIS (*n* = 53), anterior circulation AIS but no SWI data (*n* = 40), or lost to follow‐up (*n* = 38). Therefore, a total of 145 patients were included. There were no significant differences between the included and excluded patients in terms of age (62.2 ± 12.2 years versus 62.2 ± 11.9 years, *p* = .987), sex (male, 66.2% versus 75.0%, *p* = .127), NIHSS score on admission (7 [4–11] versus 6 [4–11], *p* = .391) or OTT (206.4 ± 57.1 min versus 195.2 ± 54.2 min, *p* = .391). During the study period, 24 patients with large artery occlusion were treated with direct thrombectomy; these patients had a mean age of 66.3 ± 10.3 years and a median NIHSS score on admission of 13 (11–17.75), and 16 (66.7%) of them were male. A flowchart of the selection process is shown in Figure 1.

**FIGURE 2:**
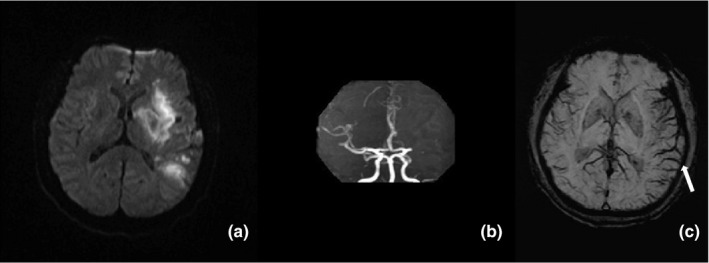
A 60‐year‐old male patient with a history of hypertension and diabetes for 5 years was admitted for AIS 3 hr after onset. He had aphasia, right hemiplegia, and an NIHSS score of 11 on admission. He was treated with IVT 4 hr after onset. An MRI scan was performed 24 hr after onset. DWI showed multi‐focal acute infarcts in the left MCA territory (a); MRA showed occlusion of the left MCA in the M1 segment (b); and SWI showed more and larger vessels with signal loss, indicating ACVS (Grade 3, white arrow) in the left MCA territory (c). His mRS was 4 at 90 days after onset, and the patient remained dependent

Brain MRI was performed within 72 hr after stroke onset. The median interval between stroke onset and MRI scanning was 22 (range, 8–58) hours. Fifty‐five (37.9%) patients had symptomatic severe ILAS or occlusion (SILASO). Thirty‐five (24.1%) patients presented with ACVS (≥Grade 1) on SWI, among whom 2 (1.8%) had Grade 1 ACVS, 18 (12.4%) had Grade 2, and 15 (10.3%) had Grade 3. All the patients with ACVS (≥Grade 1) also had SILASO, and 54.5% of patients with SILASO had ACVS (≥Grade 1). Symptomatic SILASO was highly correlated with ACVS (r = 0.559, *p* < .001). Ten patients were selected randomly to test the inter and intrarater agreement of ACVS, with kappa values of 0.75 and 0.82, respectively. A typical case with ACVS in our study is shown in Figure 2. Fifty‐three (36.6%) patients had mRS ≥ 3 at 90 days. Three died within 90 days of onset. The demographic and clinical characteristics of this study sample are shown in Table 1.

**TABLE 1:** Demographic and clinical characteristics of the study sample

Characteristics	Mean (*SD*)/Median (IQR)/*n* (%) *n* = 145
Age (years)	62.5 ± 12.2
Men (*n*, %)	96 (66.2%)
Hypertension (*n*, %)	105 (72.4%)
Diabetes mellitus (*n*, %)	40 (27.6%)
Smokers/ex‐smokers (*n*, %)	48 (33.1%)
Atrial fibrillation (*n*, %)	34 (23.4%)
Previous stroke (*n*, %)	26 (17.9%)
Previous antiplatelet therapy (*n*, %)	15 (10.3%)
OTT (min)	206.4 ± 57.1
NIHSS score on admission	7 (4–11)
BG on admission (mmol/L)	7.2 ± 2.9
SBP on admission (mm Hg)	158.7 ± 26.6
DBP on admission (mm Hg)	92.1 ± 17.3
Poor outcome (*n*, %)	53 (36.6%)
Stroke subtype	
Large artery atherosclerosis (*n*, %)	48 (33.1%)
Small artery occlusion (*n*, %)	40 (27.6%)
Cardioembolism (*n*, %)	34 (23.4%)
Other etiologies (*n*, %)	0 (0%)
Unknown etiologies (*n*, %)	23 (15.9%)
Hemorrhagic transformation (*n*, %)	40 (27.6%)
SILASO (*n*, %)	55 (37.9%)
ACVS （*n*, %）	
Grade 0	110 (75.9%)
Grade 1	2 (1.4%)
Grade 2	18 (12.4%)
Grade 3	15 (10.3%)

Abbreviations: ACVS, asymmetrical cortical vessel sign; BG, blood glucose; DBP, diastolic blood pressure; NIHSS, National Institutes of Health Stroke Scale; OTT, onset to treatment time; SBP, systolic blood pressure; SILASO, severe intracranial large artery stenosis or occlusion.

### 3.2 Univariable analysis

Compared with those with favorable outcome, patients with poor outcome were older and had higher NIHSS score on admission, more frequent previous stroke and hemorrhagic transformation, and more frequent presence of symptomatic SILASO and ACVS (≥Grade 1) (*p* < .05). The subtype of stroke based on TOAST criteria was not significantly associated with 90‐day poor outcome (Table 2).

**TABLE 2:** Comparisons of clinical and MRI variables in patients with poor and favorable outcome

	Poor outcome *N* = 53	Favorable outcome *N* = 92	*t*/*X* ^2^/*z* value	*p* Value
Age^a^ (years)	65.8 ± 10.4	60.6 ± 12,8	−2.487	.014
Men^b^ (*n*, %)	37 (69.8%)	59 (64.1%)	0.485	.486
NIHSS score on admission^c^	11 (7–14)	6 (4–9)	−4.980	<.001
Hypertension^b^ (*n*, %)	37 (69.8%)	68 (73.9%)	0.283	.595
Diabetes^b^ (*n*, %)	18 (34.0%)	22 (23.9%)	1.700	.192
Smokers/ex‐smokers^b^ (*n*, %)	15 (28.3%)	33 (35.9%)	0.870	.351
Atrial fibrillation^b^ (*n*, %)	16 (30.2%)	18 (19.6%)	2.114	.146
Previous stroke^b^ (*n*, %)	15 (28.3%)	11 (12.0%)	6.105	.013
PAT^b^ (*n*, %)	8 (15.1%)	7 (7.6%)	2.032	.154
OTT^a^ (min)	209.8 ± 53.5	204.4 ± 59.2	−0.551	.583
BG on admission^a^ (mmol/L)	7.4 ± 3.0	7.1 ± 2.3	−0.547	.585
SBP on admission^a^ (mm Hg)	153.6 ± 27.4	161.6 ± 24.2	1.805	.073
DBP on admission^a^ (mm Hg)	90.0 ± 17.1	93.3 ± 17.4	1.102	.272
Stroke subtype^b^				
LAA (*n*, %)	18 (34.0%)	30 (32.6%)	4.005	.261
SAO (*n*, %)	10 (18.9%)	30 (32.6%)		
CE (*n*, %)	16 (30.2%)	18 (19.6%)		
UE (*n*, %)	9 (17.0%)	14 (15.2%)		
HT^b^ (*n*, %)	21 (39.6%)	19 (20.7%)	6.058	.014
SILASO^b^ (*n*, %)	26 (49.1%)	29 (31.5%)	4.392	.036
ACVS^b^				
Grade 0 (*n*, %)	32 (60.4%)	78 (84.8%)	11.321	.001
Grade 1 (*n*, %)	1 (1.9%)	1 (1.1%)		
Grade 2 (*n*, %)	10 (18.9%)	8 (8.7%)		
Grade 3 (*n*, %)	10 (18.9%)	5 (5.4%)		

Abbreviations: ACVS, asymmetrical cortical vessel sign; BG, blood glucose; CE, cardiaembolism; DBP, diastolic blood pressure; HT, hemorrhagic transformation; LAA, large artery atherosclerosis; NIHSS, National Institutes of Health Stroke Scale; OTT, onset to treatment time; PAT, previous antiplatelet therapy; SAO, small artery occlusion; SBP, systolic blood pressure; SILASO, severe intracranial large artery stenosis or occlusion; UE, unknown etiologies.

^a^ Mean(*SD*),*t* test.

^b^ *n* (%), chi‐square test.

^c^ Mann–Whiteny *U* test.

### 3.3 Multivariate logistic regressions

Variables that were significantly different between the two groups in the univariable analysis were entered into subsequent logistic regression models. As symptomatic SILASO and ACVS were highly correlated, it was not appropriate to enter them into the same model at the same time. In addition, we wanted to assess the effect of classified ACVS on predictive value. Thus, we used three logistic regression models. In model 1, age, NIHSS score on admission, previous stroke, hemorrhagic transformation, and overall ACVS were entered using a backward Wald mode. Age (odds ratio [OR] = 1.040, 95% confidence interval [CI] = 1.004–1.077, *p* = .028), NIHSS score on admission (OR = 1.172, 95% CI = 1.073–1.280, *p* < .001), and ACVS (overall) (OR = 1.760, 95% CI = 1.191–2.601, *p* = .005) were significantly associated with 90‐day poor outcome. In model 2, classified ACVS rather than overall ACVS was entered, as well as the other variables listed above. Age (OR = 1.041, 95% CI = 1.005–1.079, *p* < .027), NIHSS score on admission (OR = 1.173, 95% CI = 1.074–1.281, *p* < .001), and Grade 3 ACVS (OR = 6.019, 95% CI = 1.436–25.237, *p* < .014) were significant predictors of 90‐day poor outcome. In model 3, SILASO was entered rather than ACVS. In this model, only NIHSS score on admission (OR = 1.183, 95% CI = 1.087–1.288, *p* < .001) had significance. However, symptomatic SILASO was not significantly associated with 90‐day poor outcome (*p* = .051). The multivariate logistic regression of risk factors for poor outcome is shown in Table 3.

**TABLE 3:** Multivariate logistic regressions of risk factors for poor outcome

Variable	*β*	90‐day outcome		
		OR (95% CI)	*p* Value	*R* ^2^
Model^1^				
Age	0.039	1.040 (1.004–1.077)	.028	.310
NIHSS score on admission	0.159	1.172 (1.073–1.280)	<.001	
Previous stroke	0.607	1.870 (0.655–5.399)	.242	
Hemorrhagic transformation	0.116	1.124 (0.459–2.748）	.799	
ACVS	0.565	1.760 (1.191–2.601)	.005	
Model^2^				
Age	0.040	1.041 (1.005–1.079)	.027	.311
NIHSS on admission	0.159	1.173 (1.074–1.281)	<.001	
Previous stroke	0.629	1.036 (0.999–1.075)	.240	
Hemorrhagic transformation	0.123	1.131 (0.461–2.774)	.788	
ACVS (Grade 0 as reference)			.044	
Grade 1	0.064	1.066 (0.039–29.218)	.970	
Grade 2	1.047	2.848 (0.900–9.020)	.075	
Grade 3	1.795	6.019 (1.436–25.237)	.014	
Model^3^				
Age	0.030	1.030 (0.996–1.065)	.080	.275
NIHSS on admission	0.168	1.183 (1.087–1.288)	<.001	
Previous stroke	0.585	1.794 (0.636–5.065)	.269	
Hemorrhagic transformation	0.284	1.329 (0.560–3.156)	.519	
SILASO	0.791	2.206 (0.995–4.889)	.051	

Abbreviations: ACVS, asymmetrical cortical vessel sign; NIHSS, National Institutes of Health Stroke Scale; SILASO, severe intracranial large artery stenosis or occlusion.

^1^ With overall ACVS entered.

^2^ With categorized ACVS entered.

^3^ With SILASO entered.

In patients with SILASO, 26 experienced poor outcome 90 days after onset. Among these, patients with ACVS were more likely to have a poor outcome than those without ACVS (21 [60%] versus. 5 [25%], *p* = .034). After adjustment for age, AF, history of previous stroke, and NIHSS score on admission, ACVS remained significantly associated with 90‐day poor outcome with an OR of 6.104 (*p* = .013) in patients with SILASO. The univariate and multivariate analysis results of this subgroup are shown in the supplemental materials (Tables S1–S3).

## 4 DISCUSSION

We found that the presence of ACVS was a significant predictor for 90‐day poor outcome in AIS patients treated with IVT. Among the four grades of ACVS, the most severe (Grade 3) had the most significant predictive value. As SWI is increasingly used in patients with AIS, this finding may help physicians identify those at relatively high risk of poor prognosis.

The presence of ACVS has been hypothesized to be related to increased deoxyhemoglobin (Huang et al., 2012; Kesavadas, Santhosh, & Thomas, 2010) due to decreased oxygen saturation in the hypointense vessel (An & Lin, 2000; Hermier et al., 2003; Lee et al., 2010; Xia et al., 2014). Some studies suggest that ACVS could be used as an indicator of hypoperfusion in patients with ICA stenosis (Mundiyanapurath et al., 2016) and acute thromboembolic occlusion (Baik et al., 2012). The mismatch between ACVS on SWI and lesions on DWI corresponds to the penumbra of the acute infarct (Lou et al., 2014; Wang et al., 2018). However, the utility of ACVS in predicting the prognosis of AIS patients with IVT remained unclear.

Our results show a novel finding that ACVS was a predictor of poor outcome post‐IVT at 90 days after stroke, with an OR of 1.760. In another model, Grade 3 ACVS had the most effective predictive value compared with the other grades, with an OR of 6.019. In the correlation analysis, there was no significant correlation between NIHSS score on admission and ACVS (*r* = .242), but a significant correlation was found between ACVS and symptomatic SILASO (*r* = 0.559). Thus, these two variables were not included in the same multivariate logistic regression model. ACVS was found to be associated with 90‐day poor outcome. SILASO is related to recurrence and poor outcome of AIS (WASID Study Group, 1998). In our study, all the patients with ACVS had SILASO; and in the analysis of the SILASO subgroup, ACVS was also a significant predictor 90‐day poor outcome, suggesting that the presence of ACVS might be a stronger predictor than SILASO. Good collateral circulation could contribute to favorable outcome after IVT (Cuccione, Padovano, Versace, Ferrarese, & Beretta, 2016; Fang et al., 2016; Kucinski et al., 2003; Leng, Lan, Liu, Leung, & Wong, 2016); therefore, it might also improve outcomes in patients with symptomatic SILASO who undergo IVT. Conversely, patients with ACVS on SWI may be more likely to have severe hypoperfusion and poor collateral circulation, which could result in infarction enlargement and deterioration of neurological deficits. Thus, in the presence of ACVS, the perfusion status reflected by SWI may contribute to a more comprehensive infarction assessment, which could be used to identify those with a poorer prognosis.

In our study, we only evaluated intracranial artery stenosis, which is more prevalent in non‐Caucasians (e.g., East Asians) (Gao, Wong, Huang, & Li, 2003). Therefore, our findings might be more relevant to those populations.

There were several advantages to our study. First, all participants had data including SWI, which could be directly used by clinicians without the need for complicated and time‐consuming postprocessing methods. In addition, to the best of our knowledge, it was one of the few studies that focused on the relationship between ACVS and patient outcomes after IVT. Furthermore, we used a semi‐quantitative assessment to evaluate the predictive value of ACVS. However, there were also several limitations. First, the sample size was relatively small. Second, as ACVS was only assessed once, it was not possible to examine dynamic changes. Third, computed tomography angiography or digital subtraction angiography was performed in only a few patients, potentially resulting in overestimation of intracranial stenosis and uncertainty regarding recanalization of the occluded vessels. Fourth, our findings may not be applicable for centers where advanced computed tomography imaging including angiography is more likely to be performed.

## 5 CONCLUSIONS

In conclusion, both overall ACVS and classified ACVS can be considered as useful and convenient neuroimaging markers for 90‐day poor outcome in patients with anterior circulation AIS treated with IVT, and perfusion status evaluation could play an important part in the evolution of infarction. Further prospective studies with larger sample size and repeated MR scans are warranted.

## CONFLICT OF INTEREST

None declared.

## AUTHOR CONTRIBUTIONS

YL Liu and YK Chen participated in the conception and design of the study, the analysis of clinical data, and critical revision of the manuscript for scientific validity. WM Xiao and JF Qu helped to acquire raw data. XW Fang and MQ Liang analyzed the imaging data. JK Lu, YZ Wang, ZH Lu, and HH Zhong assisted in patient follow‐ups. All authors contributed to data analysis, drafting or revising the article, gave final approval of the version to be published, and agree to be accountable for all aspects of the work.

## Supporting information

Tables S1–S3

## Data Availability

The data that support the findings of this study are available from the corresponding author upon reasonable request.
