# Supplementary material for: Asymmetrical cortical vessel sign predicts prognosis after acute ischemic stroke
Source: Brain Behav. 2020 May 20;10(7):e01657. doi: 10.1002/brb3.1657 (PMC7375089; doi:10.1002/brb3.1657)
Supplement: Supplementary file 1 — Tables S1–S3 [file BRB3-10-e01657-s001.docx]

Table S1. Comparisons of demographic and clinical characteristics in patients with or without ACVS in SILASO (+) group

|  | With ACVS  N=35 | Without ACVS  N=20 | *P* Value |
| --- | --- | --- | --- |
| Age^a^ (years) | 61.0±13.8 | 64.9±10.9 | 0.268 |
| Men^b^ (n,%) | 25(71.4%) | 16(80%) | 0.472 |
| NIHSS score on admission^c^ | 8(6-14.8) | 10(5-14) | 0.798 |
| Hypertension^b^ (n,%) | 20(57.1%) | 17(85%) | 0.374 |
| Diabetes^b^ (n,%) | 7(20.0%) | 5(25.0%) | 0.990 |
| Smokers/ex-smokers^b^ (n,%) | 12(34.3%) | 8(40.0%) | 0.836 |
| Atrial fibrillation^b^ (n,%) | 10(28.6%) | 3(15.0%) | 0.198 |
| Previous stroke^b^ (n,%) | 8(22.9%) | 4(20.0%) | 0.742 |
| PAT^b^ (n,%) | 5(14.3%) | 2(10.0%) | 0.686 |
| OTT^a^ (minutes) | 203.8±58.6 | 223.8±46.1 | 0.179 |
| BG on admission^a^ (mmol/L) | 7.0±2.0 | 7.0±2.8 | 0.936 |
| SBP on admission^a^ (mmHg) | 150.9±20.4 | 155.5±23.1 | 0.452 |
| DBP on admission^a^ (mmHg) | 90.2±16.1 | 88.5±16.8 | 0.717 |
| Hemorrhagic transformation^b^ (n,%) | 13(37.1%) | 5(25.0%) | 0.178 |
| 90-day poor outcome^b^ (n,%) | 21(60.0%) | 5(25.0%) | 0.034 |

Note: a Mean(SD),t-test; b n(%), chi-square test; c, mann-whiteny U test

ACVS=asymmetrical cortical vessel sign; BG=blood glucose; DBP= diastolic blood pressure; NIHSS=National Institutes of Health Stroke Scale; OTT=onset to treatment time; PAT= previous antiplatelet therapy; SBP= Systolic blood pressure; SILASO= severe intracranial large artery stenosis or occlusion;

Table S2. Comparisons of clinical and MRI variables in patients with poor and favorable outcome in SILASO (+) group

|  | Poor outcome  N=26 | Favorable outcome  N=29 | *P* Value |
| --- | --- | --- | --- |
| Age^a^ (years) | 67.6±9.6 | 58.2±13.7 | 0.005 |
| Men^b^ (n,%) | 21(80.8%) | 20(69.0%) | 0.316 |
| NIHSS score on admission^c^ | 12(7-15.3) | 8(4-11.5%) | 0.006 |
| Hypertension^b^ (n,%) | 18(69.2%) | 19(65.5%) | 0.769 |
| Diabetes^b^ (n,%) | 9(34.6%) | 3(10.3%) | 0.061 |
| Smokers/ex-smokers^b^ (n,%) | 10(38.5%) | 10(34.5%) | 0.759 |
| Atrial fibrillation^b^ (n,%) | 10(38.5%) | 3(10.3%) | 0.024 |
| Previous stroke^b^ (n,%) | 10(38.5%) | 2(6.9%) | 0.008 |
| PAT^b^ (n,%) | 6(23.1%) | 1(3.4%) | 0.053 |
| OTT^a^ (minutes) | 207.6±56.4 | 216.3±52.7 | 0.559 |
| BG on admission^a^ (mmol/L) | 7.2±2.3 | 6.8±2.3 | 0.561 |
| SBP on admission^a^ (mmHg) | 153.0±22.6 | 152.7±21.0 | 0.959 |
| DBP on admission^a^ (mmHg) | 88.4±14.3 | 90.4±17.9 | 0.659 |
| Hemorrhagic transformation^b^ (n,%) | 12(46.2%) | 6(20.7%) | 0.080 |
| ACVS^b^ (n,%) | 21(80.8%) | 14(48.3%) | 0.034 |

Note: a Mean(SD),t-test; b n(%), chi-square test; c, mann-whiteny U test

ACVS=asymmetrical cortical vessel sign; BG=blood glucose; DBP= diastolic blood pressure; NIHSS=National Institutes of Health Stroke Scale; OTT=onset to treatment time; PAT= previous antiplatelet therapy; SBP= Systolic blood pressure; SILASO= severe intracranial large artery stenosis or occlusion;

Table S3. Multivariate logistic regressions of risk factors for 90-day poor outcome in SILASO (+) group

| Variable | 90-day poor outcome | |
| --- | --- | --- |
|  | *OR*（95% *CI*） | *P* value |
| Age | 1.083 (1.015-1.155) | 0.016 |
| NIHSS score on admission | 1.142 (0.992-1.314) | 0.064 |
| Atrial fibrillation | 2.904 (0.484-17.409) | 0.243 |
| Previous stroke | 3.943 (0.668-23.289) | 0.130 |
| ACVS | 6.104 (1.471-25.317) | 0.013 |

ACVS=asymmetrical cortical vessel sign; NIHSS=National Institutes of Health Stroke Scale; SILASO= severe intracranial large artery stenosis or occlusion;
